# Association of Stressful Life Events With Oral Health Among Japanese Workers

**DOI:** 10.2188/jea.JE20220225

**Published:** 2024-01-05

**Authors:** Jin Aoki, Takashi Zaitsu, Akiko Oshiro, Jun Aida

**Affiliations:** Department of Oral Health Promotion, Graduate School of Medical and Dental Sciences, Tokyo Medical and Dental University, Tokyo, Japan

**Keywords:** stressful life events, oral health, occupational health, occupational dentistry, augmented inverse-probability weighting

## Abstract

**Background:**

Psychological stress can cause various mental and physical health problems. The previous results on stress and oral health are inconsistent, possibly because of the narrow stress measurements. We aimed to examine the association between a broader range of stressful life events and oral health among workers.

**Methods:**

This cross-sectional study analyzed anonymous individual data from a national survey in Japan. Data on stressful life events, oral health problems which are one or more of tooth pain, gum swelling/bleeding, and difficulty chewing, and covariates were obtained using a self-reported questionnaire. Covariates used included gender, age group, and disease under treatment. Logistic regression analysis was used to estimate the association between stressful life events and oral health problems. We then estimated the causal treatment effects of stress using the augmented inverse-probability weighting (AIPW) method.

**Results:**

Among the 274,881 subjects, 152,850 men (55.6%) and 122,031 women (44.4%) with a mean age of 47.0 (standard deviation, 14.4) years, 4.0% reported oral health problems, with a prevalence of 2.1% among those without any stress. The prevalence increased with stress score, reaching 15.4% for those with the maximum stress score. The adjusted odds ratio of this group compared to those without any stress was 9.2 (95% confidence interval [CI], 8.2–10.3). The estimated prevalence of oral health problems by the AIPW analysis was 2.2% (95% CI, 2.1–2.3%) for those without any stress and 14.4% (95% CI, 12.1–16.7%) for those with the maximum stress scores.

**Conclusion:**

There was a clear dose-response association between stressful life events and oral health problems.

## INTRODUCTION

Psychological stress causes various mental and physical health problems.^1^^,^^2^ Studies have reported that chronic exposure to stress can increase the risk of developing mental health disorders and dementia.^3^^,^^4^ Physically, stress increases blood pressure, the risk of stroke, and cardiovascular disease.^5^^–^^7^ In addition, the effects of stress on the immune system can increase upper respiratory tract infections.^8^ Psychological stress is also a mechanism linking social inequalities and health.^9^^,^^10^

There is a possibility that psychological stress increases the risk of oral diseases. From a biomedical perspective, stress influences the progression of periodontal disease through its effect on immune capacity and disruption of the bone remodeling balance.^11^ A positive correlation has been observed between stress-related biomarkers and the severity of periodontal disease.^11^ Another study reported that emotional stress is one of the risk factors for bruxism, and persistent bruxism negatively affects oral health.^12^ Daily stress may also make it difficult to take appropriate oral health behaviors in one’s daily life.^13^ Epidemiological studies reported the association between current psychological stress and poor oral health.^14^ A recent study reported the association of worsened socioeconomic conditions due to COVID-19 with dental pain, and psychological stress mediated this association.^15^ However, another study reported no significant association between work stress and primary oral health conditions like dental caries and tooth loss.^16^ This inconsistency in results may be explained by stress measurements. The stress measurements in previous studies do not necessarily encompass a broader range of stress. In addition, the dose-response association between stress and oral health has not been examined.

In Japan, there is an urgent need to prevent stress-related health problems. The number of industrial accidents resulting in brain and neurological diseases caused by overwork and mental disorders was a record high in 2020.^17^ However, oral health problems caused by stress have not been considered in industrial settings. Major dental diseases such as dental caries and periodontal disease developed by the long-term accumulation of risk over the life course. Stress from life events would affect lifestyle and oral environment and accumulate the risk of dental diseases over the life course, which would increase oral problems. This study aimed to determine the association between a broader range of stressful life events and oral health among workers in Japan. We hypothesized that individuals with higher numbers of stressful life events would have a larger number of subjective oral symptoms.

## METHODS

**Figure 1.**
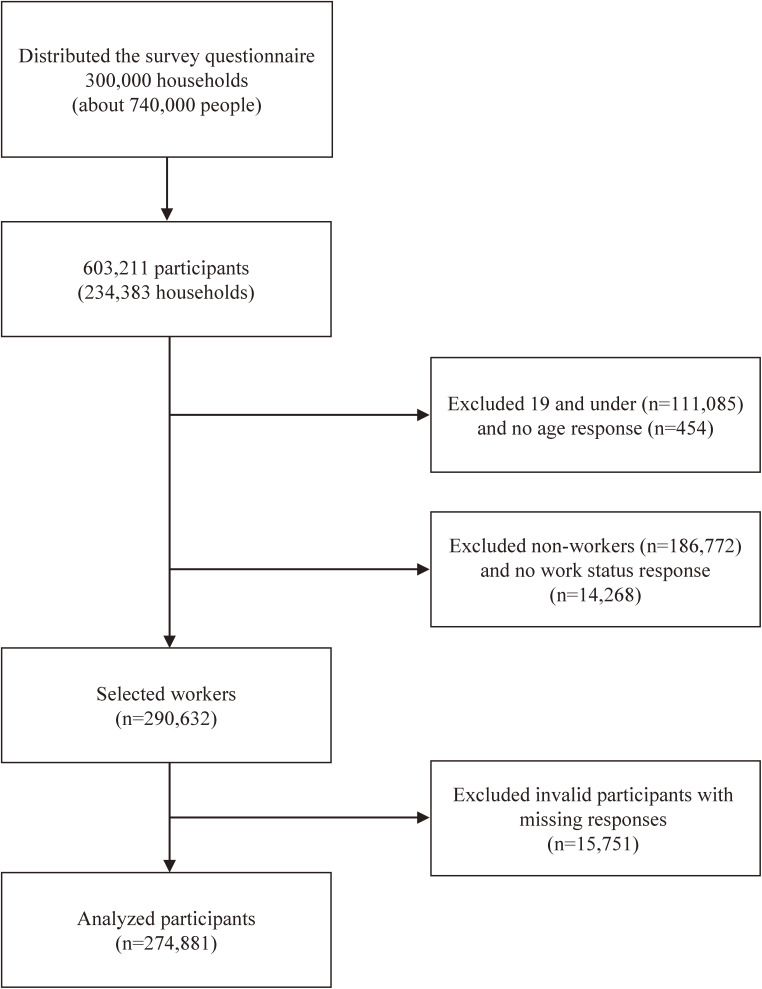
Process of extracting analysis targets from all respondents of the survey

This cross-sectional study used anonymous individual data from the 2013 Comprehensive Survey of Living Conditions in Japan, a national survey conducted by the Japanese government. From the 5,530 areas selected by stratified random selection, all household members (approximately 740,000 people) from 300,000 households were distributed in the survey questionnaire. In total, 603,211 participants (234,383 households) responded to the questionnaire. To include workers in our analysis, we first excluded 111,085 participants who were aged 19 years or younger and 454 participants who did not provide an age response. We then excluded 186,772 participants who were non-workers and 14,268 participants who did not provide a work status response. After excluding 15,751 invalid participants with missing responses, data from 274,881 participants were included in the analysis (Figure 1).

The survey was conducted by the Ministry of Health, Labour and Welfare of Japan, and under Article 36 of the Statistical Act, we obtained permission for this secondary data analysis. The ethical review committee did not deem the review necessary for the secondary analysis of anonymized data.

### Oral health problems

Data about oral health problems were collected with the question, “Do you have anything wrong (subjective symptoms) such as sickness or injury in the last few days?” Participants were asked to select any symptoms from 42 choices relating to symptoms of the eyes, ears, chest, respiratory system, digestive system, teeth, skin, musculoskeletal system, limbs, urogenital system, injuries, and others. Among the 42 choices, three were about oral health problems: tooth pain, gum swelling/bleeding, and difficulty chewing. We defined those with oral health problems as those who selected one or more oral health problems.

### Stress score

Previous studies examined the association of a broader range of stressful life events with physical and mental illness, and the associations of the number of stressful life events and illness have been reported.^18^^–^^21^ The survey asked whether there is any current stress and the following question was used to grasp stressful life events and stressors: “Do you currently have worries or stress in your daily life?”. Participants were asked to select any choices from 1) relationship with family; 2) relationships with people other than family; 3) things about love and sex; 4) marriage; 5) divorce; 6) bullying, sexual harassment; 7) regarding the purpose of life; 8) I do not have free time; 9) income, household budget, debt; 10) my illness and long-term care; 11) family illness and long-term care; 12) pregnancy and childbirth; 13) childcare; 14) housework; 15) own studies, entrance exams, and going on to higher education; 16) children’s education; 17) own work; 18) family’s work; 19) living and living environment; and 20) other. To examine the dose-response association between this wide range of stressful life events and oral health, following previous studies,^20^^,^^21^ we counted the number of choices each participant selected and used this number as the stress score. When calculating this score, since the analysis was conducted on oral health in this study, the response to “10) my illness and long-term care” was excluded from the score.

### Covariates

As the covariates, we used gender, age, marital status, occupational classification, educational background, smoking habits, drinking habits, health activities, medical checkups, a variable for whether they could do their usual activities due to health problems, and presence of disease under treatment.

Age was classified as 20–29, 30–39, 40–49, 50–59, and ≥60 years. Marital status was classified as currently married, never married, or divorced or widowed. Occupations were classified into white-collar workers, blue-collar workers, and other, while educational background was classified as more than a vocational school or high school. Smoking habits were classified as never smoking, quit smoking, or smoking, and drinking habits were classified as never drinking, quit drinking, or drinking. Health activities were classified as either executed or non-executed. Medical checkups were classified as either consulted or not consulted. Health status was also adjusted by two variables. The variable of whether they could perform their usual activities due to health problems was classified as none and unable to perform their usual activities. For the variable of the presence of disease under treatment, participants were asked to select the diseases under treatment from 42 choices and also asked to choose the most important disease for the participant. In our analysis, we used the 14 categories of diseases which were provided by the questionnaire: 1) endocrine and metabolic disorders, 2) psychiatric and neurological diseases, 3) eye diseases, 4) ear diseases, 5) cardiovascular system disease, 6) respiratory system disease, 7) digestive system disease, 8) skin disease, 9) musculoskeletal system disease, 10) urinary and reproductive system disease, 11) injury, 12) anemia and blood diseases, 13) malignant neoplasm (cancer), and 14) other diseases. When participants did not have any diseases or selected dental disease, they were categorized as none. Further adjustment of the disease, symptoms, and health behaviors were not conducted because there was a possibility that they worked as a mediator between stress and oral health. For example, it is possible that stress increases diabetes, and diabetes increases the risk of periodontal disease. In this case, diabetes becomes a mediator between stress and periodontal disease. Mediator adjustment is not recommended when examining the total effects of stress on periodontal disease.^22^ Since the stress score in this study excluded the stress from health problems, systemic health condition is considered as mediator rather than confounder. Therefore, we did not adjust for any further systemic health variables.

### Statistical analysis

A directed acyclic graph is shown in eFigure 1. From the distribution of the stress score, scores of 7 or more were combined into one category. Then, we cross-tabulated each variable according to the presence or absence of oral health problems. To compare the association of each variable with the presence of oral health problems, logistic regression analysis was used to estimate the univariable and multivariable odds ratios for oral health problems. Finally, we estimate the causal treatment effects of stress using the augmented inverse-probability weighting (AIPW) method. AIPW is a “doubly robust” method that makes a robust estimate when an outcome (oral health problems) regression model or a propensity score model for estimating the probability of having stress is correctly specified.^23^ We used AIPW combined inverse-probability weighted methods using “*teffects aipw*” command of Stata software. Covariates were used to calculate the propensity score and the outcome logistic regression model. The results were described by the potential outcome means. For sensitivity analysis, we also conducted analyses using the presence of each oral health problem as the outcome. In addition, to test the validity of the results, we added another analysis: there was a possibility of a reporting bias that stressed workers were more likely to overreport oral problems to the self-reported questionnaire. Therefore, we examined whether stressed workers are more likely to attend dental clinics. Descriptive and multivariable logistic regression analyses were used to determine the association between stress and dental attendance. All analyses were performed using Stata/MP version 17 software (Stata Corp., College Station, TX, USA). The level of statistical significance was set at *P* < 0.05.

## RESULTS

There were 152,850 men (55.6%) and 122,031 women (44.4%) with a mean age of 47.0 (standard deviation [SD], 14.4) years. The mean and median stress scores in this study were 1.0 (SD, 1.5) and 0, and the distribution is shown in eFigure 2. The descriptive distribution of oral health problems according to the stress score and oral health problems are shown in Table 1 and eTable 1, respectively. The distribution of each oral health problem for the sensitivity analysis are shown in eTable 2. In total, 4.0% of participants answered that they had oral health problems. Among those without any stress (score = 0), only 2.1% reported any oral health problems. However, the percentage increased by stress score, reaching 15.4% for those with the maximum stress score (score = 7 or more). This 15.4% was the highest prevalence compared with the prevalence of other characteristics we considered. Participants who reported “could not do usual activities due to health problems” had the second-highest prevalence of oral health problems (14.1%). The prevalence of oral health problems among those 60 years old or older and those who stopped drinking was 6.6% and 6.7%, respectively.

**Table 1.** Descriptive distribution of the number of stressful life events (*n* = 274,881)

		*n* (%)	Number of stressful life events (%)							
			0	1	2	3	4	5	6	7 or more
Total		274,881 (100.0)								
Oral health problems	No	263,787 (96.0)	97.9	95.5	93.5	92.8	91.2	89.8	88.0	84.6
	Yes	11,094 (4.0)	2.1	4.5	6.5	7.2	8.8	10.2	12.0	15.4
Symptoms of tooth pain	No	269,864 (98.2)	99.0	98.1	97.2	96.9	96.1	94.7	94.0	91.9
	Yes	5,017 (1.8)	1.0	1.9	2.8	3.1	3.9	5.3	6.0	8.1
Symptoms of gum swelling/bleeding	No	270,069 (98.2)	99.2	98.1	97.0	96.7	95.7	95.4	94.2	91.9
	Yes	4,812 (1.8)	0.8	1.9	3.0	3.3	4.3	4.6	5.8	8.1
Symptoms of difficulty in chewing	No	271,814 (98.9)	99.4	98.8	98.3	98.1	97.7	97.4	96.7	95.7
	Yes	3,067 (1.1)	0.6	1.2	1.7	1.9	2.3	2.6	3.3	4.3
Attending a hospital or clinic due to dental disease	No	262,157 (95.4)	96.2	95.0	94.2	93.8	94.2	93.7	93.3	93.6
	Yes	12,724 (4.6)	3.8	5.0	5.8	6.2	5.8	6.3	6.7	6.4
Gender	Male	152,850 (55.6)	60.8	54.9	49.9	45.1	39.8	38.6	37.7	38.5
	Female	122,031 (44.4)	39.2	45.1	50.1	54.9	60.2	61.4	62.3	61.5
Age group, years	20–29	36,685 (13.3)	13.6	13.0	12.4	13.1	14.1	14.6	15.3	15.6
	30–39	55,236 (20.1)	18.7	20.3	20.2	22.6	25.1	26.8	29.2	33.1
	40–49	63,578 (23.1)	20.8	23.5	25.5	28.3	28.9	31.6	31.1	31.4
	50–59	58,974 (21.5)	20.0	23.2	24.7	23.4	22.0	19.9	18.9	16.1
	≥60	60,408 (22.0)	26.9	20.1	17.2	12.7	9.9	7.0	5.5	3.8
Marital status	Currently married	185,915 (67.6)	67.8	68.8	67.4	66.0	65.4	65.1	65.5	65.8
	Never married	64,686 (23.5)	24.0	22.6	22.8	23.6	23.8	24.4	24.4	23.3
	Divorced/widowed	24,280 (8.8)	8.3	8.6	9.8	10.4	10.9	10.4	10.1	10.9
Occupational classification	White-collar worker	185,132 (67.3)	64.1	70.1	70.5	72.1	73.3	74.9	73.7	73.4
	Blue-collar worker	66,588 (24.2)	26.9	21.6	22.0	20.6	19.5	17.8	18.9	18.6
	Other	23,161 (8.4)	9.0	8.3	7.5	7.3	7.3	7.4	7.4	8.0
Educational background	More than a vocational school	135,035 (49.1)	51.5	46.8	47.8	45.7	43.9	43.5	43.2	43.1
	Until high school	139,846 (50.9)	48.5	53.2	52.2	54.3	56.1	56.5	56.8	56.9
Smoking habits	Never smoking	183,452 (66.7)	66.7	66.5	66.6	66.6	68.8	68.0	66.2	67.0
	Quitting smoking	14,950 (5.4)	4.9	5.7	6.2	6.7	6.2	6.6	6.8	7.3
	Smoking	76,479 (27.8)	28.4	27.8	27.2	26.7	25.0	25.5	26.9	25.7
Drinking habits	Never drinking	129,997 (47.3)	47.3	46.5	47.1	48.1	48.9	49.6	48.8	49.0
	Quitting drinking	3,526 (1.3)	1.2	1.3	1.4	1.5	1.3	1.7	1.8	2.2
	Drinking	141,358 (51.4)	51.5	52.3	51.5	50.4	49.9	48.7	49.4	48.9
Health activities	Execution	227,466 (82.8)	83.2	82.4	82.5	82.0	82.5	81.8	80.5	79.3
	No execution	47,415 (17.2)	16.8	17.6	17.5	18.0	17.5	18.2	19.5	20.7
Medical checkups	Consulted	199,249 (72.5)	71.6	74.3	73.7	73.2	71.8	71.1	69.8	70.0
	Not consulted	75,632 (27.5)	28.4	25.7	26.3	26.8	28.2	28.9	30.2	30.0
Whether or not they could do their usual activities due to health problems	None	252,626 (91.9)	95.8	89.6	87.6	86.4	85.0	82.3	78.4	75.8
	Unable to do their usual activities	22,255 (8.1)	4.2	10.4	12.4	13.6	15.0	17.7	21.6	24.2
Disease under treatment	None	190,541 (69.3)	72.9	64.6	63.7	66.9	67.4	67.2	65.8	67.1
	Endocrine and metabolic disorders	12,834 (4.7)	4.5	5.2	5.3	4.3	3.5	3.6	3.7	3.4
	Psychiatric and neurological disease	3,650 (1.3)	0.6	1.6	2.1	2.4	3.2	4.1	5.0	4.7
	Eye diseases	2,977 (1.1)	1.0	1.3	1.2	1.2	1.1	0.9	0.9	0.7
	Ear diseases	812 (0.3)	0.2	0.4	0.4	0.3	0.4	0.2	0.3	0.4
	Cardiovascular system disease	20,992 (7.6)	8.1	8.0	7.7	6.2	5.3	4.6	3.9	4.0
	Respiratory system disease	4,647 (1.7)	1.3	1.9	2.2	2.3	2.3	2.5	2.5	2.6
	Digestive system disease	4,529 (1.6)	1.4	2.0	2.1	1.8	1.7	1.9	1.8	1.6
	Skin disease	4,091 (1.5)	1.1	1.8	2.0	1.9	2.4	2.3	2.9	2.2
	Musculoskeletal system disease	14,441 (5.3)	4.3	6.5	6.5	6.4	6.2	5.9	5.6	5.4
	Urinary and reproductive system disease	2,360 (0.9)	0.8	0.9	0.9	0.8	0.8	0.7	0.5	1.0
	Injury	1,751 (0.6)	0.5	0.8	0.8	0.6	0.6	0.8	0.8	1.1
	Anemia and blood diseases	790 (0.3)	0.2	0.3	0.4	0.3	0.5	0.6	0.5	0.4
	Malignant neoplasm (cancer)	1,192 (0.4)	0.3	0.5	0.6	0.5	0.4	0.5	0.7	0.4
	Other diseases	9,274 (3.4)	2.8	3.9	4.2	4.1	4.3	4.1	5.0	5.1

Table 2 shows the logistic regression results on the association between the stress score and the presence of oral health problems. In univariate analysis, compared to participants without any stress (score = 0), those with the highest stress score (score = 7 or more) showed 8.7 (95% confidence interval [CI), 7.8–9.7) times higher odds of having oral health problems. After adjusting for gender, age group, marital status, occupational classification, educational background, smoking habits, drinking habits, health activities, medical checkups, whether they could do their usual activities due to health problems, and disease under treatment, the odds ratio of those with a score of 7 or more was still significant (OR 9.2; 95% CI, 8.2–10.3). Those with scores of 1–6 also showed significantly higher odds ratios. These odds ratios were higher than those for almost all other variables (eTable 4). As the sensitivity analyses, when oral health problems, tooth pain, gum swelling/bleeding, and difficulty chewing were analyzed separately, the results were consistent (eTable 5, eTable 6, and eTable 7).

**Table 2.** Results of logistic regression on the association between stress score and presence of oral health problems (*n* = 274,881)

		Univariable analysis				Multivariable analysis			

		Odds ratio	95% CI		*P*-value	Odds ratio	95% CI		*P*-value
			Lower	Upper			Lower	Upper	
Number of stressful life events	0	1				1			
	1	2.2	2.1	2.3	*P* < 0.001	2.1	2.0	2.2	*P* < 0.001
	2	3.3	3.1	3.5	*P* < 0.001	3.1	2.9	3.3	*P* < 0.001
	3	3.7	3.4	3.9	*P* < 0.001	3.7	3.4	3.9	*P* < 0.001
	4	4.6	4.2	5.0	*P* < 0.001	4.8	4.4	5.2	*P* < 0.001
	5	5.4	4.9	6.0	*P* < 0.001	5.7	5.1	6.3	*P* < 0.001
	6	6.5	5.7	7.4	*P* < 0.001	6.7	5.8	7.6	*P* < 0.001
	7 or more	8.7	7.8	9.7	*P* < 0.001	9.2	8.2	10.3	*P* < 0.001

Other analyses have confirmed that stressed workers are more likely to attend dental clinics (eTable 3 and eTable 8). These results suggest that stressed workers do not exaggerate their responses to the questionnaire but actually have problems requiring dental visits.

**Figure 2.**
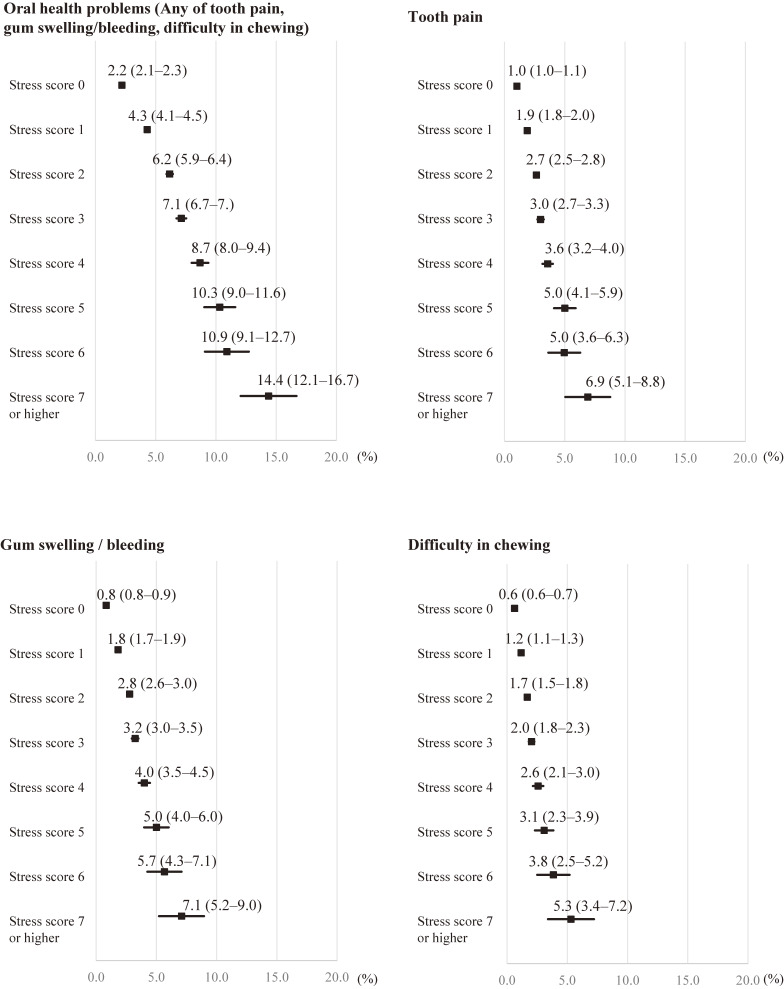
Estimated prevalence (%) of oral health problems by stress score obtained from the augmented inverse-probability weighting^*^ ^*^Considering gender, age group, marital status, occupational classification, educational background, smoking habits, drinking habits, health activities, medical checkups, a variable for whether they could do their usual activities due to health problems, and presence of disease under treatment.

Figure 2 shows the estimated prevalence of oral health problems according to each stress score using the AIPW method. Dose-response associations were observed. Generally, as the stress score increased, oral health problems increased. When each component of oral health problems was analyzed separately, similar dose-response associations were observed.

## DISCUSSION

This study examined the association between a broader range of stressful life events and oral health among workers. There was a clear dose-response association between stress scores and oral health problems. As stress scores increased, oral health problems also increased. This association between stress and oral health problems was stronger than between stress and smoking, which is a significant risk factor for oral diseases. Our study also confirmed that stressed workers had oral health problems that required dental visits.

The present results are consistent with those of previous studies that examined the association between stress and oral health.^11^^,^^12^^,^^14^^,^^24^^–^^26^ Our study adds to the knowledge that a broader range of stressful life events is associated with oral health in a dose-response manner. Although our analyzed population consisted of workers, stress outside work, such as financial and child-related stress, was also associated with the presence of oral problems. These results suggest that a wide variety of everyday events can cause stress, which increases the risk of oral health problems. A previous study suggested that worsened economic conditions due to the COVID-19 pandemic increased stress, deteriorating oral health.^15^

There were possible mechanisms explaining the association between stressful life events and oral health problems. As mentioned in the introduction, stress possibly causes periodontal disease due to the disruption of immune capacity.^11^ Stress also causes bruxism, and bruxism is a risk factor for temporomandibular disorder.^12^^,^^27^ Because temporomandibular disorder often causes pain around an ear, patients sometimes attend an otolaryngologist before they are diagnosed with temporomandibular disorder. In our analysis, those who chose ear disease as the disease under treatment also had significantly higher odds of having oral problems (eTable 4).

This study highlights the need to consider stressful life events as risk factors for poor oral health. Therefore, measures to reduce these stresses are required. In Japan, stress checks are currently being performed on workers as a component of the workplace health promotion policy, and industrial physicians are required to intervene in cases where severe stress is detected. In addition to this type of secondary prevention, the intervention aims to change the social determinants of stress as primary prevention. For example, social support is known to buffer stress.^28^ Social support is obtained from social relationships, and an intervention that aims to increase social relationships reports a positive health effect.^29^ As bi-directional relationships between oral health and social interaction have been reported,^30^ there is a possibility that an increase in social relationships improves oral health through stress reduction.

The strengths of this study include the fact that it considered a wide range of stressful life events outside work among workers. In addition, the large number of participants (*n* = 274,881) enabled representative sample analysis from all of Japan and the use of the AIPW causal inference method. These points enabled this study to show the dose-response association between stress and oral health. The first limitation of this study was that the measurements were responses to a self-reported questionnaire. However, they are subject to non-differential misclassification, which would attenuate the observed association. In addition, our analysis using dental attendance as the outcome supported the validity of the association. The cross-sectional design is another limitation of temporal relationships. However, it seems unlikely that dental problems would increase the variety of stressful life events, such as work and marital problems. In addition, this study examined the association of life events that might have mid- or long-term effects with recent oral problems. Therefore, exposure was likely to have occurred prior to the occurrence of the recent outcome. From these points, it is reasonable to assume that oral problems increased due to stress. Another shortcoming is that the stress score is calculated by simply adding up the number of stressful life events and does not consider the exposure period of the stress. A method, such as weighting from a regression analysis of individual scores, could be considered. Such a method would make the association between stress and oral health stronger. However, this study does not appear to be overestimating the association of stressful life events with oral health problems. Also, for purposes such as people counting their stress in their daily lives, a simple scoring method such as that used in this study would be more feasible than more complex assessment methods.

### Conclusion

There was a clear dose-response association between stressful life events and oral health problems. Further studies are required to determine the underlying mechanisms and develop interventions to improve oral health through stress reduction.

## SUPPLEMENTARY MATERIAL

eTable 1. Descriptive distribution of oral health problems (n = 274,881)

eTable 2. Descriptive distribution of symptoms of tooth pain, gum swelling/bleeding, and difficulty in chewing (n = 274,881)

eTable 3. Descriptive distribution of patients attending a hospital or clinic due to dental disease (n = 274,881)

eTable 4. Logistic regression analysis of the association between stress score and the presence of oral health problems (n = 274,881)

eTable 5. Logistic regression analysis of the association between stress scores and tooth pain symptoms (n = 274,881)

eTable 6. Logistic regression analysis of the association between stress score and gumswelling/bleeding symptoms (n = 274,881)

eTable 7. Logistic regression analysis of the association between stress scores and chewing difficulty symptoms (n = 274,881)

eTable 8. Logistic regression analysis of the association between the stress score and attending a hospital or clinic due to dental disease (n = 274,881)

eFigure 1. Directed acyclic graph for the association between stressful life events and oral health problems

eFigure 2. Distribution of the stress score
