# Supplementary material for: Association of Stressful Life Events With Oral Health Among Japanese Workers
Source: J Epidemiol. 2024 Jan 5;34(1):16–22. doi: 10.2188/jea.JE20220225 (PMC10701250; doi:10.2188/jea.JE20220225)
Supplement: Supplementary file 1 [file je-34-016-s001.pdf]

eTable 1. Descriptive distribution of oral health problems (n=274,881)

eTable 2. Descriptive distribution of symptoms of tooth pain, gum swelling/bleeding, and difficulty in chewing (n=274,881)

eTable 3. Descriptive distribution of patients attending a hospital or clinic due to dental disease (n=274,881)

eTable 4. Logistic regression analysis of the association between stress score and the presence of oral health problems (n=274,881)

eTable 5. Logistic regression analysis of the association between stress scores and tooth pain symptoms (n=274,881)

eTable 6. Logistic regression analysis of the association between stress score and gum-swelling/bleeding symptoms (n=274,881)

eTable 7. Logistic regression analysis of the association between stress scores and chewing difficulty symptoms (n=274,881)

eTable 8. Logistic regression analysis of the association between the stress score and attending a hospital or clinic due to dental disease (n=274,881)

eFigure 1. Directed acyclic graph for the association between stressful life events and oral health problems

eFigure 2. Distribution of the stress score

eTable 1. Descriptive distribution of oral health problems (n=274,881)

		n (%)	Oral health problems (%)	
			No	Yes
Total		274,881 (100.0)	96.0	4.0
Number of stressful life events	0	147,328 (53.6)	97.9	2.1
	1	55,600 (20.2)	95.5	4.5
	2	32,655 (11.9)	93.5	6.5
	3	19,550 (7.1)	92.8	7.2
	4	9,594 (3.5)	91.2	8.8
	5	4,925 (1.8)	89.8	10.2
	6	2,412 (0.9)	88.0	12.0
	7 or more	2,817 (1.0)	84.6	15.4
Gender	Male	152,850 (55.6)	96.1	3.9
	Female	122,031 (44.4)	95.8	4.2
Age group, years	20–29	36,685 (13.3)	97.5	2.5
	30–39	55,236 (20.1)	97.2	2.8
	40–49	63,578 (23.1)	96.9	3.1
	50–59	58,974 (21.5)	95.4	4.6
	≥60	60,408 (22.0)	93.4	6.6
Marital status	Currently married	185,915 (67.6)	95.8	4.2
	Never married	64,686 (23.5)	97.0	3.0
	Divorced/ widowed	24,280 (8.8)	94.3	5.7

Occupational classification	White-collar worker	185,132 (67.3)	96.2	3.8
	Blue-collar worker	66,588 (24.2)	95.6	4.4
	Other	23,161 (8.4)	95.3	4.7
Educational background	More than a vocational school	135,035 (49.1)	95.5	4.5
	Until high school	139,846 (50.9)	96.4	3.6
Smoking habits	Never smoking	183,452 (66.7)	96.2	3.8
	Quitting smoking	14,950 (5.4)	94.2	5.8
	Smoking	76,479 (27.8)	95.8	4.2
Drinking habits	Never drinking	129,997 (47.3)	96.0	4.0
	Quitting drinking	3,526 (1.3)	93.3	6.7
	Drinking	141,358 (51.4)	96.0	4.0
Health activities	Execution	227,466 (82.8)	96.0	4.0
	No execution	47,415 (17.2)	96.0	4.0
Medical checkups	Consulted	199,249 (72.5)	96.1	3.9
	Not consulted	75,632 (27.5)	95.5	4.5
Whether or not they could do their usual activities due to health problems	None	252,626 (91.9)	96.9	3.1

	Unable to do their usual activities	22,255 (8.1)	85.9	14.1
Disease under treatment	None	190,541 (69.3)	97.1	2.9
	Endocrine and metabolic disorders	12,834 (4.7)	94.0	6.0
	Psychiatric and neurological disease	3,650 (1.3)	90.9	9.1
	Eye diseases	2,977 (1.1)	91.5	8.5
	Ear diseases	812 (0.3)	90.8	9.2
	Cardiovascular system disease	20,992 (7.6)	94.3	5.7
	Respiratory system disease	4,647 (1.7)	94.1	5.9
	Digestive system disease	4,529 (1.6)	92.1	7.9
	Skin disease	4,091 (1.5)	95.3	4.7
	Musculoskeletal system disease	14,441 (5.3)	92.3	7.7
	Urinary and reproductive system disease	2,360 (0.9)	91.9	8.1
	Injury	1,751 (0.6)	93.7	6.3
	Anemia and blood diseases	790 (0.3)	92.2	7.8

	Malignant neoplasm (cancer)	1,192 (0.4)	91.7	8.3
	Other diseases	9,274 (3.4)	93.5	6.5

eTable 2. Descriptive distribution of symptoms of tooth pain, gum swelling/bleeding, and difficulty in chewing (n=274,881)

		n (%)	symptoms of tooth pain		symptoms of gum swelling / bleeding		symptoms of difficulty in chewing	
			No	Yes	No	Yes	No	Yes
Total		274,881 (100.0)	98.2	1.8	98.2	1.8	98.9	1.1
Number of stressful life events	0	147,328 (53.6)	99.0	1.0	99.2	0.8	99.4	0.6
	1	55,600 (20.2)	98.1	1.9	98.1	1.9	98.8	1.2
	2	32,655 (11.9)	97.2	2.8	97.0	3.0	98.3	1.7
	3	19,550 (7.1)	96.9	3.1	96.7	3.3	98.1	1.9
	4	9,594 (3.5)	96.1	3.9	95.7	4.3	97.7	2.3
	5	4,925 (1.8)	94.7	5.3	95.4	4.6	97.4	2.6
	6	2,412 (0.9)	94.0	6.0	94.2	5.8	96.7	3.3
	7 or more	2,817 (1.0)	91.9	8.1	91.9	8.1	95.7	4.3
Gender	Male	152,850 (55.6)	98.2	1.8	98.4	1.6	98.9	1.1
	Female	122,031 (44.4)	98.2	1.8	98.1	1.9	98.9	1.1

Age group, years	20–29	36,685 (13.3)	98.3	1.7	99.1	0.9	99.8	0.2
	30–39	55,236 (20.1)	98.2	1.8	98.9	1.1	99.8	0.2
	40–49	63,578 (23.1)	98.3	1.7	98.6	1.4	99.5	0.5
	50–59	58,974 (21.5)	98.2	1.8	97.6	2.4	98.8	1.2
	≥60	60,408 (22.0)	97.9	2.1	97.4	2.6	97.0	3.0
Marital status	Currently married	185,915 (67.6)	98.2	1.8	98.2	1.8	98.8	1.2
	Never married	64,686 (23.5)	98.3	1.7	98.8	1.2	99.5	0.5
	Divorced/ widowed	24,280 (8.8)	97.8	2.2	97.5	2.5	97.9	2.1
Occupational classification	White-collar worker	185,132 (67.3)	98.2	1.8	98.3	1.7	99.1	0.9
	Blue-collar worker	66,588 (24.2)	98.1	1.9	98.2	1.8	98.5	1.5
	Other	23,161 (8.4)	98.0	2.0	98.0	2.0	98.4	1.6

Educational background	More than a vocational school	135,035 (49.1)	98.1	1.9	98.1	1.9	98.6	1.4
	Until high school	139,846 (50.9)	98.3	1.7	98.4	1.6	99.2	0.8
Smoking habits	Never smoking	183,452 (66.7)	98.3	1.7	98.3	1.7	98.9	1.1
	Quitting smoking	14,950 (5.4)	97.5	2.5	97.3	2.7	98.4	1.6
	Smoking	76,479 (27.8)	97.9	2.1	98.3	1.7	98.9	1.1
Drinking habits	Never drinking	129,997 (47.3)	98.2	1.8	98.3	1.7	98.9	1.1
	Quitting drinking	3,526 (1.3)	97.3	2.7	97.0	3.0	97.5	2.5
	Drinking	141,358 (51.4)	98.2	1.8	98.3	1.7	98.9	1.1
Health activities	Execution	227,466 (82.8)	98.2	1.8	98.2	1.8	98.9	1.1
	No execution	47,415 (17.2)	97.9	2.1	98.3	1.7	99.0	1.0
Medical checkups	Consulted	199,249 (72.5)	98.2	1.8	98.3	1.7	99.0	1.0

	Not consulted	75,632 (27.5)	98.0	2.0	98.1	1.9	98.6	1.4
Whether or not they could do their usual activities due to health problems	None	252,626 (91.9)	98.5	1.5	98.6	1.4	99.3	0.7
	Unable to do their usual activities	22,255 (8.1)	94.3	5.7	93.8	6.2	94.5	5.5
Disease under treatment	None	190,541 (69.3)	98.5	1.5	98.8	1.2	99.4	0.6
	Endocrine and metabolic disorders	12,834 (4.7)	97.9	2.1	97.2	2.8	98.0	2.0
	Psychiatric and neurological disease	3,650 (1.3)	96.4	3.6	95.3	4.7	97.4	2.6
	Eye diseases	2,977 (1.1)	96.9	3.1	96.4	3.6	96.7	3.3
	Ear diseases	812 (0.3)	97.0	3.0	97.2	2.8	95.9	4.1

Cardiovascular system disease	20,992 (7.6)	98.1	1.9	97.4	2.6	98.0	2.0
Respiratory system disease	4,647 (1.7)	97.3	2.7	97.7	2.3	98.0	2.0
Digestive system disease	4,529 (1.6)	97.0	3.0	96.2	3.8	97.5	2.5
Skin disease	4,091 (1.5)	97.8	2.2	97.9	2.1	98.9	1.1
Musculoskeletal system disease	14,441 (5.3)	97.1	2.9	96.8	3.2	97.1	2.9
Urinary and reproductive system disease	2,360 (0.9)	96.7	3.3	97.3	2.7	96.8	3.2
Injury	1,751 (0.6)	97.1	2.9	97.9	2.1	97.8	2.2
Anemia and blood diseases	790 (0.3)	97.3	2.7	96.7	3.3	96.7	3.3

	Malignant neoplasm (cancer)	1,192 (0.4)	96.9	3.1	95.7	4.3	97.5	2.5
	Other diseases	9,274 (3.4)	97.3	2.7	97.0	3.0	98.1	1.9

eTable 3. Descriptive distribution of patients attending a hospital or clinic due to dental disease (n=274,881)

		n (%)	Attending a hospital or clinic due to dental disease	
			No	Yes
Total		274,881 (100.0)	95.4	4.6
Number of stressful life events	0	147,328 (53.6)	96.2	3.8
	1	55,600 (20.2)	95.0	5.0
	2	32,655 (11.9)	94.2	5.8
	3	19,550 (7.1)	93.8	6.2
	4	9,594 (3.5)	94.2	5.8
	5	4,925 (1.8)	93.7	6.3
	6	2,412 (0.9)	93.3	6.7
	7 or more	2,817 (1.0)	93.6	6.4
Gender	Male	152,850 (55.6)	95.7	4.3
	Female	122,031 (44.4)	94.9	5.1
Age group, years	20–29	36,685 (13.3)	97.1	2.9
	30–39	55,236 (20.1)	96.3	3.7
	40–49	63,578 (23.1)	95.8	4.2
	50–59	58,974 (21.5)	94.8	5.2
	≥60	60,408 (22.0)	93.5	6.5
Marital status	Currently married	185,915 (67.6)	95.2	4.8
	Never married	64,686 (23.5)	96.3	3.7
	Divorced/widowed	24,280 (8.8)	94.5	5.5

Occupational classification	White-collar worker	185,132 (67.3)	95.3	4.7
	Blue-collar worker	66,588 (24.2)	95.6	4.4
	Other	23,161 (8.4)	95.0	5.0
Educational background	More than a vocational school	135,035 (49.1)	95.2	4.8
	Until high school	139,846 (50.9)	95.6	4.4
Smoking habits	Never smoking	183,452 (66.7)	95.3	4.7
	Quitting smoking	14,950 (5.4)	93.8	6.2
	Smoking	76,479 (27.8)	95.7	4.3
Drinking habits	Never drinking	129,997 (47.3)	95.4	4.6
	Quitting drinking	3,526 (1.3)	95.0	5.0
	Drinking	141,358 (51.4)	95.4	4.6
Health activities	Execution	227,466 (82.8)	95.2	4.8
	No execution	47,415 (17.2)	96.2	3.8
Medical checkups	Consulted	199,249 (72.5)	95.3	4.7
	Not consulted	75,632 (27.5)	95.7	4.3
Whether or not they could do their usual activities due to health problems	None	252,626 (91.9)	95.6	4.4
	Unable to do their usual activities	22,255 (8.1)	92.5	7.5
Disease under treatment	None	190,541 (69.3)	95.9	4.1
	Endocrine and metabolic disorders	12,834 (4.7)	94.2	5.8

Psychiatric and neurological disease	3,650 (1.3)	94.4	5.6
Eye diseases	2,977 (1.1)	91.0	9.0
Ear diseases	812 (0.3)	93.1	6.9
Cardiovascular system disease	20,992 (7.6)	94.8	5.2
Respiratory system disease	4,647 (1.7)	94.7	5.3
Digestive system disease	4,529 (1.6)	94.6	5.4
Skin disease	4,091 (1.5)	94.0	6.0
Musculoskeletal system disease	14,441 (5.3)	94.6	5.4
Urinary and reproductive system disease	2,360 (0.9)	94.2	5.8
Injury	1,751 (0.6)	96.7	3.3
Anemia and blood diseases	790 (0.3)	94.7	5.3
Malignant neoplasm (cancer)	1,192 (0.4)	93.4	6.6
Other diseases	9,274 (3.4)	92.1	7.9

eTable 4. Logistic regression analysis of the association between stress score and the presence of oral health problems (n=274,881)

		Univariable analysis				Multivariable analysis			
		Odds ratio	95% CI		*P*-value	Odds ratio	95% CI		*P*-value
			Lower	Upper			Lower	Upper	
Number of stressful life events	0	1				1			
	1	2.2	2.1	2.3	*P*<0.001	2.1	2.0	2.2	*P*<0.001
	2	3.3	3.1	3.5	*P*<0.001	3.1	2.9	3.3	*P*<0.001
	3	3.7	3.4	3.9	*P*<0.001	3.7	3.4	3.9	*P*<0.001
	4	4.6	4.2	5.0	*P*<0.001	4.8	4.4	5.2	*P*<0.001
	5	5.4	4.9	6.0	*P*<0.001	5.7	5.1	6.3	*P*<0.001
	6	6.5	5.7	7.4	*P*<0.001	6.7	5.8	7.6	*P*<0.001
	7 or more	8.7	7.8	9.7	*P*<0.001	9.2	8.2	10.3	*P*<0.001
Gender	Male	1				1			
	Female	1.1	1.1	1.1	*P*<0.001	1.0	1.0	1.1	0.073
Age group, years	20–29	1				1			
	30–39	1.1	1.0	1.2	0.007	1.0	0.9	1.1	0.978

	40–49	1.3	1.2	1.4	*P*<0.001	1.1	1.0	1.2	0.123
	50–59	1.9	1.7	2.0	*P*<0.001	1.6	1.5	1.8	*P*<0.001
	≥60	2.8	2.6	3.0	*P*<0.001	2.7	2.4	2.9	*P*<0.001
Marital status	Currently married	1				1			
	Never married	0.7	0.7	0.7	*P*<0.001	1.0	1.0	1.1	0.185
	Divorced/widowed	1.4	1.3	1.5	*P*<0.001	1.1	1.0	1.1	0.122
Occupational classification	White-collar worker	1				1			
	Blue-collar worker	1.2	1.1	1.2	*P*<0.001	1.0	1.0	1.1	0.060
	Other	1.2	1.2	1.3	*P*<0.001	1.1	1.0	1.1	0.057
Educational background	More than a vocational school	1				1			
	Until high school	0.8	0.8	0.8	*P*<0.001	0.9	0.9	1.0	*P*<0.001
Smoking habits	Never smoking	1				1			
	Quitting smoking	1.6	1.4	1.7	*P*<0.001	1.5	1.3	1.6	*P*<0.001
	Smoking	1.1	1.0	1.1	*P*<0.001	1.3	1.2	1.3	*P*<0.001

Drinking habits	Never drinking	1				1			
	Quitting drinking	1.7	1.5	2.0	*P*<0.001	1.1	0.9	1.2	0.271
	Drinking	1.0	1.0	1.0	0.656	1.0	1.0	1.1	0.101
Health activities	Execution	1				1			
	No execution	1.0	1.0	1.1	0.987	1.1	1.1	1.2	*P*<0.001
Medical checkups	Consulted	1				1			
	Not consulted	1.2	1.1	1.2	*P*<0.001	1.1	1.1	1.2	*P*<0.001
Whether or not they could do their usual activities due to health problems	None	1				1			
	Unable to do their usual activities	5.1	4.9	5.3	*P*<0.001	2.7	2.6	2.9	*P*<0.001
Disease under treatment	None	1				1			
	Endocrine and metabolic disorders	2.2	2.0	2.3	*P*<0.001	1.4	1.2	1.5	*P*<0.001

Psychiatric and neurological disease	3.4	3.0	3.8	*P*<0.001	1.3	1.1	1.5	*P*<0.001
Eye diseases	3.1	2.7	3.6	*P*<0.001	1.8	1.5	2.0	*P*<0.001
Ear diseases	3.4	2.7	4.4	*P*<0.001	1.8	1.4	2.3	*P*<0.001
Cardiovascular system disease	2.1	1.9	2.2	*P*<0.001	1.3	1.2	1.3	*P*<0.001
Respiratory system disease	2.1	1.9	2.4	*P*<0.001	1.4	1.2	1.6	*P*<0.001
Digestive system disease	2.9	2.6	3.3	*P*<0.001	1.7	1.5	1.9	*P*<0.001
Skin disease	1.7	1.4	1.9	*P*<0.001	1.3	1.1	1.5	0.003
Musculoskeletal system disease	2.8	2.6	3.0	*P*<0.001	1.4	1.3	1.5	*P*<0.001
Urinary and reproductive system disease	3.0	2.5	3.4	*P*<0.001	1.5	1.3	1.8	*P*<0.001
Injury	2.3	1.9	2.8	*P*<0.001	1.0	0.8	1.3	0.708

Anemia and blood diseases	2.9	2.2	3.7	*P*<0.001	1.9	1.4	2.5	*P*<0.001
Malignant neoplasm (cancer)	3.1	2.5	3.8	*P*<0.001	1.4	1.1	1.7	0.002
Other diseases	2.3	2.1	2.6	*P*<0.001	1.4	1.3	1.5	*P*<0.001

CI, confidence interval.

eTable 5. Logistic regression analysis of the association between stress scores and tooth pain symptoms (n=274,881)

		Univariable analysis				Multivariable analysis			
		Odds ratio	95%CI Lower	95%CI Upper	*P*-value	Odds ratio	95%CI Lower	95%CI Upper	*P*-value
Number of stressful life events	0	1				1			
	1	2.0	1.9	2.2	*P*<0.001	1.9	1.7	2.1	*P*<0.001
	2	2.9	2.7	3.2	*P*<0.001	2.7	2.5	2.9	*P*<0.001
	3	3.3	3.0	3.6	*P*<0.001	3.0	2.8	3.4	*P*<0.001
	4	4.2	3.7	4.7	*P*<0.001	3.9	3.5	4.4	*P*<0.001
	5	5.8	5.0	6.6	*P*<0.001	5.3	4.6	6.1	*P*<0.001
	6	6.5	5.5	7.8	*P*<0.001	5.7	4.8	6.8	*P*<0.001
	7 or more	9.1	7.9	10.5	*P*<0.001	7.8	6.7	9.1	*P*<0.001
Gender	Male	1				1			
	Female	1.0	1.0	1.1	0.426	1.0	0.9	1.0	0.184
Age group, years	20–29	1				1			
	30–39	1.1	1.0	1.2	0.319	1.0	0.9	1.1	0.425

	40–49	1.0	0.9	1.1	0.729	0.9	0.8	1.0	0.014
	50–59	1.1	1.0	1.2	0.179	1.0	0.9	1.1	0.470
	≥60	1.3	1.2	1.4	*P*<0.001	1.3	1.1	1.4	*P*<0.001
Marital status	Currently married	1				1			
	Never married	1.0	0.9	1.0	0.328	1.1	1.0	1.1	0.210
	Divorced/ widowed	1.2	1.1	1.4	p<0.001	1.0	0.9	1.1	0.562
Occupational classification	White-collar worker	1				1			
	Blue-collar worker	1.1	1.0	1.2	0.004	1.1	1.0	1.1	0.092
	Other	1.1	1.0	1.2	0.014	1.1	1.0	1.2	0.271

Educational background	More than a vocational school	1				1			
	Until high school	0.9	0.8	0.9	*P*<0.001	0.9	0.9	1.0	0.064
Smoking habits	Never smoking	1				1			
	Quitting smoking	1.5	1.3	1.7	*P*<0.001	1.4	1.2	1.5	*P*<0.001
	Smoking	1.3	1.2	1.4	*P*<0.001	1.3	1.2	1.4	*P*<0.001
Drinking habits	Never drinking	1				1			
	Quitting drinking	1.5	1.2	1.9	*P*<0.001	1.0	0.8	1.3	0.681
	Drinking	1.0	1.0	1.1	0.741	1.0	0.9	1.1	0.802
Health activities	Execution	1				1			

	No execution	1.2	1.1	1.3	*P*<0.001	1.2	1.1	1.3	*P*<0.001
Medical checkups	Consulted	1				1			
	Not consulted	1.2	1.1	1.2	*P*<0.001	1.1	1.0	1.2	0.008
Whether or not they could do their usual activities due to health problems	None	1				1			
	Unable to do their usual activities	4.0	3.7	4.3	*P*<0.001	2.6	2.4	2.8	*P*<0.001
Disease under treatment	None	1				1			
	Endocrine and metabolic disorders	1.398	1.2	1.6	*P*<0.001	1.1	1.0	1.3	0.071
	Psychiatric and neurological disease	2.441	2.0	2.9	*P*<0.001	1.0	0.8	1.2	0.770
	Eye diseases	2.075	1.7	2.6	*P*<0.001	1.5	1.2	1.9	*P*<0.001
	Ear diseases	1.982	1.3	3.0	0.001	1.3	0.8	1.9	0.248

Cardiovascular system disease	1.29	1.2	1.4	*P*<0.001	1.1	1.0	1.2	0.285
Respiratory system disease	1.799	1.5	2.2	*P*<0.001	1.3	1.1	1.5	0.009
Digestive system disease	1.984	1.7	2.4	*P*<0.001	1.4	1.1	1.7	0.001
Skin disease	1.43	1.2	1.8	0.001	1.1	0.9	1.3	0.564
Musculoskeletal system disease	1.949	1.8	2.2	*P*<0.001	1.2	1.1	1.3	0.002
Urinary and reproductive system disease	2.224	1.8	2.8	*P*<0.001	1.5	1.2	1.9	0.001
Injury	1.952	1.5	2.6	*P*<0.001	1.0	0.8	1.4	0.932
Anemia and blood diseases	1.777	1.2	2.7	0.010	1.3	0.8	2.0	0.285
Malignant neoplasm (cancer)	2.084	1.5	2.9	*P*<0.001	1.2	0.9	1.7	0.210

	Other diseases	1.825	1.6	2.1	P<0.001	1.2	1.1	1.4	0.001

eTable 6. Logistic regression analysis of the association between stress score and gum-swelling/bleeding symptoms (n=274,881)

		Univariable analysis				Multivariable analysis			
		Odds ratio	95% CI		*P*-value	Odds ratio	95% CI		*P*-value
			Lower	Upper			Lower	Upper	
Number of stressful life events	0	1				1			
	1	2.5	2.3	2.7	*P*<0.001	2.3	2.1	2.5	*P*<0.001
	2	3.9	3.6	4.3	*P*<0.001	3.6	3.3	3.9	*P*<0.001
	3	4.5	4.1	4.9	*P*<0.001	4.2	3.8	4.7	*P*<0.001
	4	5.8	5.1	6.5	*P*<0.001	5.6	5.0	6.3	*P*<0.001
	5	6.2	5.3	7.1	*P*<0.001	6.1	5.2	7.1	*P*<0.001
	6	7.9	6.6	9.5	*P*<0.001	7.6	6.3	9.2	*P*<0.001
	7 or more	11.3	9.8	13.1	*P*<0.001	11.3	9.7	13.2	*P*<0.001
Gender	Male	1				1			
	Female	1.2	1.1	1.3	*P*<0.001	1.1	1.0	1.2	0.005
Age group, years	20–29	1				1			
	30–39	1.3	1.1	1.5	*P*<0.001	1.1	1.0	1.3	0.163

	40–49	1.6	1.4	1.8	*P*<0.001	1.3	1.2	1.5	*P*0.001
	50–59	2.7	2.4	3.0	*P*<0.001	2.2	1.9	2.5	*P*<0.001
	≥60	3.0	2.6	3.3	*P*<0.001	2.8	2.4	3.2	*P*<0.001
Marital status	Currently married	1				1			
	Never married	0.6	0.6	0.7	*P*<0.001	1.0	0.9	1.1	0.981
	Divorced/widowed	1.4	1.3	1.5	*P*<0.001	1.1	1.0	1.2	0.209
Occupational classification	White-collar worker	1				1			
	Blue-collar worker	1.1	1.0	1.1	0.152	1.0	0.9	1.1	0.578
	Other	1.2	1.1	1.3	0.001	1.0	0.9	1.2	0.370
Educational background	More than a vocational school	1				1			
	Until high school	0.8	0.8	0.8	*P*<0.001	0.9	0.8	1.0	0.001
Smoking habits	Never smoking	1				1			
	Quitting smoking	1.6	1.5	1.8	*P*<0.001	1.5	1.4	1.7	*P*<0.001
	Smoking	1.0	0.9	1.1	0.986	1.1	1.1	1.2	*P*<0.001

Drinking habits	Never drinking	1				1			
	Quitting drinking	1.7	1.4	2.1	p<0.001	1.1	0.9	1.3	0.392
	Drinking	1.0	0.9	1.1	0.836	1.0	1.0	1.1	0.267
Health activities	Execution	1				1			
	No execution	1.0	0.9	1.1	0.968	1.1	1.1	1.2	0.001
Medical checkups	Consulted	1				1			
	Not consulted	1.1	1.0	1.2	0.001	1.1	1.0	1.2	0.012
Whether or not they could do their usual activities due to health problems	None	1				1			
	Unable to do their usual activities	4.8	4.5	5.1	*P*<0.001	2.5	2.3	2.7	*P*<0.001
Disease under treatment	None	1				1			
	Endocrine and metabolic disorders	2.4	2.1	2.7	*P*<0.001	1.5	1.3	1.7	*P*<0.001
	Psychiatric and neurological disease	4.0	3.4	4.7	*P*<0.001	1.5	1.3	1.8	*P*<0.001

Eye diseases	3.0	2.5	3.7	*P*<0.001	1.7	1.4	2.1	*P*<0.001
Ear diseases	2.4	1.6	3.6	*P*<0.001	1.2	0.8	1.9	0.345
Cardiovascular system disease	2.2	2.0	2.4	*P*<0.001	1.4	1.2	1.5	*P*<0.001
Respiratory system disease	1.9	1.6	2.3	*P*<0.001	1.2	1.0	1.5	0.093
Digestive system disease	3.2	2.8	3.8	*P*<0.001	1.9	1.6	2.2	*P*<0.001
Skin disease	1.7	1.4	2.1	*P*<0.001	1.3	1.0	1.6	0.020
Musculoskeletal system disease	2.7	2.4	3.0	*P*<0.001	1.3	1.2	1.5	*P*<0.001
Urinary and reproductive system disease	2.3	1.8	2.9	*P*<0.001	1.2	0.9	1.5	0.240
Injury	1.8	1.3	2.4	0.001	0.8	0.6	1.1	0.221
Anemia and blood diseases	2.8	1.9	4.1	*P*<0.001	1.7	1.1	2.5	0.012

	Malignant neoplasm (cancer)	3.6	2.7	4.8	$P<0.001$	1.6	1.2	2.2	0.001
	Other diseases	2.5	2.2	2.8	$P<0.001$	1.5	1.3	1.7	$P<0.001$

CI, confidence interval.

eTable 7. Logistic regression analysis of the association between stress scores and chewing difficulty symptoms (n=274,881)

		Univariable analysis				Multivariable analysis			
		Odds ratio	95% CI		*P*-value	Odds ratio	95% CI		*P*-value
			Lower	Upper			Lower	Upper	
Number of stressful life events	0	1				1			
	1	2.1	1.9	2.3	*P*<0.001	2.0	1.8	2.2	*P*<0.001
	2	2.9	2.6	3.2	*P*<0.001	2.9	2.6	3.2	*P*<0.001
	3	3.2	2.8	3.6	*P*<0.001	3.7	3.3	4.2	*P*<0.001
	4	3.8	3.3	4.4	*P*<0.001	4.8	4.1	5.7	*P*<0.001
	5	4.3	3.6	5.2	*P*<0.001	6.0	4.9	7.3	*P*<0.001
	6	5.7	4.5	7.1	*P*<0.001	8.3	6.5	10.6	*P*<0.001
	7 or more	7.5	6.1	9.0	*P*<0.001	12.2	9.9	15.1	*P*<0.001
Gender	Male	1				1			
	Female	1.0	0.9	1.1	0.699	1.0	0.9	1.1	0.833
Age group, years	20–29	1				1			
	30–39	0.9	0.7	1.2	0.435	0.8	0.6	1.1	0.123

	40–49	2.1	1.7	2.6	P<0.001	1.8	1.4	2.3	P<0.001
	50–59	4.9	3.9	6.0	P<0.001	4.3	3.4	5.4	P<0.001
	≥60	12.5	10.1	15.4	P<0.001	12.1	9.5	15.3	P<0.001
Marital status	Currently married	1				1			
	Never married	0.4	0.4	0.5	P<0.001	1.1	1.0	1.3	0.054
	Divorced/widowed	1.8	1.6	1.9	P<0.001	1.2	1.1	1.3	0.003
Occupational classification	White-collar worker	1				1			
	Blue-collar worker	1.6	1.5	1.7	P<0.001	1.1	1.1	1.3	0.002
	Other	1.8	1.6	2.0	P<0.001	1.2	1.1	1.4	0.002
Educational background	More than a vocational school	1				1			
	Until high school	0.6	0.5	0.6	P<0.001	0.9	0.8	0.9	P<0.001
Smoking habits	Never smoking	1				1			
	Quitting smoking	1.5	1.3	1.7	P<0.001	1.4	1.2	1.6	P<0.001
	Smoking	1.1	1.0	1.2	0.063	1.4	1.3	1.5	P<0.001

Drinking habits	Never drinking	1				1			
	Quitting drinking	2.3	1.8	2.8	*P*<0.001	1.2	1.0	1.5	0.125
	Drinking	1.0	0.9	1.1	0.982	1.0	1.0	1.1	0.418
Health activities	Execution	1				1			
	No execution	0.8	0.8	0.9	0.001	1.1	1.0	1.3	0.014
Medical checkups	Consulted	1				1			
	Not consulted	1.4	1.3	1.6	*P*<0.001	1.3	1.2	1.4	*P*<0.001
Whether or not they could do their usual activities due to health problems	None	1				1			
	Unable to do their usual activities	8.0	7.4	8.6	*P*<0.001	3.4	3.1	3.7	*P*<0.001
Disease under treatment	None	1				1			
	Endocrine and metabolic disorders	3.4	3.0	3.9	*P*<0.001	1.4	1.2	1.6	*P*<0.001

Psychiatric and neurological disease	4.4	3.6	5.5	*P*<0.001	1.4	1.1	1.8	0.003
Eye diseases	5.6	4.5	6.9	*P*<0.001	1.9	1.5	2.4	*P*<0.001
Ear diseases	7.0	4.9	9.9	*P*<0.001	2.5	1.7	3.6	*P*<0.001
Cardiovascular system disease	3.3	3.0	3.7	*P*<0.001	1.2	1.1	1.4	0.003
Respiratory system disease	3.3	2.7	4.1	*P*<0.001	1.7	1.4	2.1	*P*<0.001
Digestive system disease	4.2	3.4	5.1	*P*<0.001	1.6	1.3	1.9	*P*<0.001
Skin disease	1.7	1.3	2.4	*P*<0.001	1.4	1.0	1.9	0.051
Musculoskeletal system disease	4.8	4.3	5.4	*P*<0.001	1.5	1.4	1.7	*P*<0.001
Urinary and reproductive system disease	5.5	4.3	6.9	*P*<0.001	1.6	1.3	2.1	*P*<0.001
Injury	3.7	2.7	5.2	*P*<0.001	1.2	0.9	1.7	0.247

	Anemia and blood diseases	5.6	3.8	8.3	P<0.001	3.1	2.1	4.7	P<0.001
	Malignant neoplasm (cancer)	4.2	2.9	6.1	P<0.001	1.2	0.8	1.8	0.329
	Other diseases	3.2	2.7	3.7	P<0.001	1.5	1.2	1.7	P<0.001

CI, confidence interval.

eTable 8. Logistic regression analysis of the association between the stress score and attending a hospital or clinic due to dental disease (n=274,881)

		Univariable analysis				Multivariable analysis			
		Odds ratio	95% CI		*P*-value	Odds ratio	95% CI		*P*-value
			Lower	Upper			Lower	Upper	
Number of stressful	0	1				1			
life events	1	1.3	1.3	1.4	*P*<0.001	1.3	1.3	1.4	*P*<0.001
	2	1.6	1.5	1.7	*P*<0.001	1.5	1.5	1.6	*P*<0.001
	3	1.7	1.6	1.8	*P*<0.001	1.7	1.6	1.8	*P*<0.001
	4	1.6	1.4	1.7	*P*<0.001	1.6	1.5	1.8	*P*<0.001
	5	1.7	1.5	1.9	*P*<0.001	1.8	1.6	2.0	*P*<0.001
	6	1.8	1.5	2.1	*P*<0.001	1.9	1.6	2.2	*P*<0.001
	7 or more	1.7	1.5	2.0	*P*<0.001	1.8	1.6	2.2	*P*<0.001
Gender	Male	1				1			
	Female	1.2	1.2	1.2	*P*<0.001	1.2	1.1	1.2	*P*<0.001
Age group, years	20–29	1	1	1		1			
	30–39	1.3	1.2	1.4	*P*<0.001	1.3	1.2	1.4	*P*<0.001

	40–49	1.5	1.4	1.6	$P<0.001$	1.5	1.4	1.6	$P<0.001$
	50–59	1.8	1.7	2.0	$P<0.001$	1.9	1.7	2.0	$P<0.001$
	≥60	2.3	2.2	2.5	$P<0.001$	2.6	2.4	2.8	$P<0.001$
Marital status	Currently married	1				1			
	Never married	0.8	0.7	0.8	$P<0.001$	1.1	1.0	1.2	0.001
	Divorced/widowed	1.1	1.1	1.2	$P<0.001$	1.0	0.9	1.1	0.754
Occupational classification	White-collar worker	1				1			
	Blue-collar worker	0.9	0.9	1.0	0.004	0.9	0.9	1.0	$P<0.001$
	Other	1.1	1.0	1.1	0.053	1.0	0.9	1.1	0.782
Educational background	More than a vocational school	1				1			
	Until high school	0.9	0.9	1.0	$P<0.001$	1.0	0.9	1.0	0.416
Smoking habits	Never smoking	1				1			
	Quitting smoking	1.3	1.3	1.4	$P<0.001$	1.4	1.3	1.5	$P<0.001$
	Smoking	0.9	0.9	0.9	$P<0.001$	1.1	1.0	1.1	0.004

Drinking habits	Never drinking	1				1			
	Quitting drinking	1.1	0.9	1.3	0.213	0.9	0.8	1.1	0.402
	Drinking	1.0	1.0	1.0	0.617	1.0	1.0	1.1	0.065
Health activities	Execution	1				1			
	No execution	0.8	0.8	0.8	*P*<0.001	0.9	0.8	0.9	*P*<0.001
Medical checkups	Consulted	1				1			
	Not consulted	0.9	0.9	0.9	*P*<0.001	0.9	0.9	0.9	*P*<0.001
Whether or not they could do their usual activities due to health problems	None	1				1			
	Unable to do their usual activities	1.8	1.7	1.9	*P*<0.001	1.3	1.3	1.4	*P*<0.001
Disease under treatment	None	1				1			
	Endocrine and metabolic disorders	1.5	1.3	1.6	*P*<0.001	1.1	1.0	1.1	0.189
	Psychiatric and neurological disease	1.4	1.2	1.6	*P*<0.001	1.0	0.9	1.2	0.771

Eye diseases	2.3	2.0	2.6	*P*<0.001	1.6	1.4	1.8	*P*<0.001
Ear diseases	1.7	1.3	2.3	*P*<0.001	1.2	0.9	1.6	0.120
Cardiovascular system disease	1.3	1.2	1.4	*P*<0.001	0.9	0.9	1.0	0.017
Respiratory system disease	1.3	1.2	1.5	*P*<0.001	1.1	0.9	1.2	0.342
Digestive system disease	1.3	1.2	1.5	*P*<0.001	1.0	0.9	1.1	0.922
Skin disease	1.5	1.3	1.7	*P*<0.001	1.4	1.2	1.6	*P*<0.001
Musculoskeletal system disease	1.4	1.3	1.5	*P*<0.001	1.0	0.9	1.0	0.266
Urinary and reproductive system disease	1.4	1.2	1.7	*P*<0.001	1.0	0.8	1.2	0.951
Injury	0.8	0.6	1.0	0.106	0.6	0.5	0.8	*P*<0.001
Anemia and blood diseases	1.3	1.0	1.8	0.081	1.0	0.8	1.4	0.814

	Malignant neoplasm (cancer)	1.7	1.3	2.1	P<0.001	1.1	0.9	1.4	0.462
	Other diseases	2.0	1.9	2.2	P<0.001	1.6	1.5	1.7	P<0.001

CI, confidence interval.

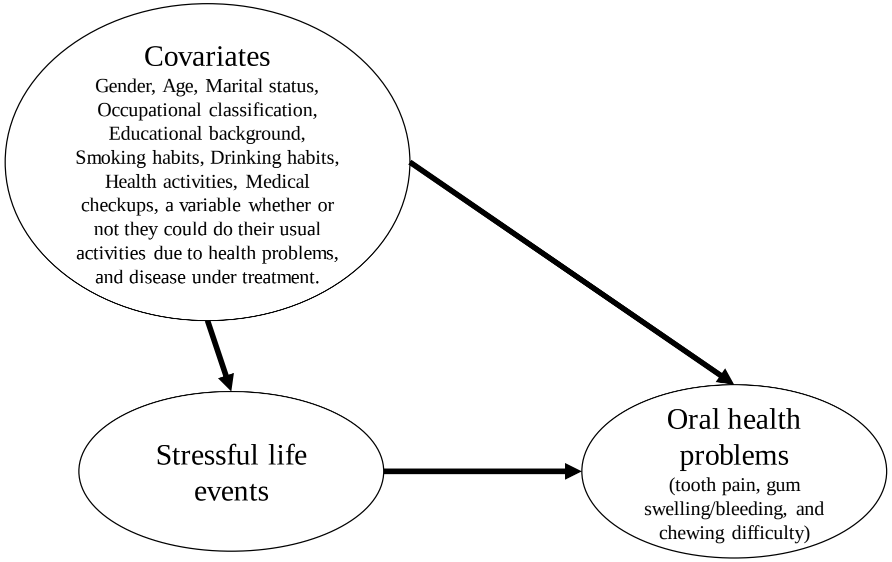

eFigure 1. Directed acyclic graph for the association between stressful life events and oral health problems

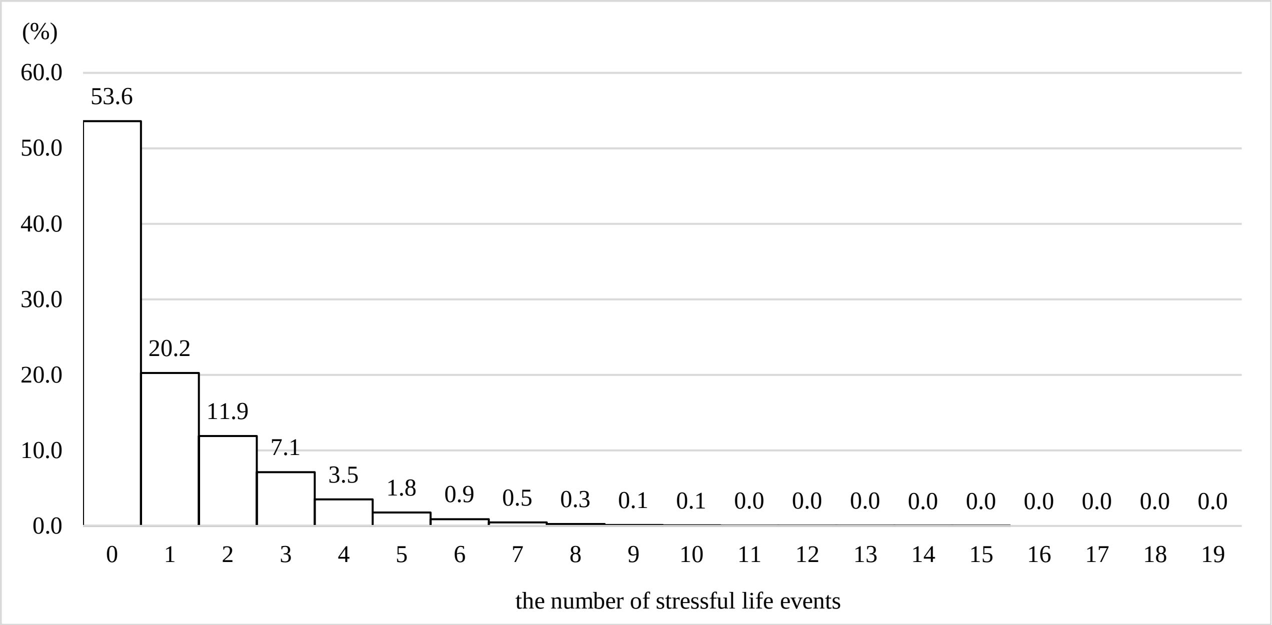

eFigure 2. Distribution of the stress score
